# Effect of COVID-19 Pandemic on Hepatocellular Carcinoma Diagnosis: Results from a Tertiary Care Center in North-West Italy

**DOI:** 10.3390/curroncol29030119

**Published:** 2022-02-24

**Authors:** Davide Giuseppe Ribaldone, Gian Paolo Caviglia, Silvia Gaia, Emanuela Rolle, Alessandra Risso, Daniela Campion, Paola Rita Brunocilla, Giorgio Maria Saracco, Patrizia Carucci

**Affiliations:** 1 Department of Medical Sciences, University of Turin, 10100 Turin, Italy; gianpaolo.caviglia@unito.it (G.P.C.); alessandrarisso82@gmail.com (A.R.); giorgiomaria.saracco@unito.it (G.M.S.); 2 Division of Gastroenterology, Città della Salute e della Scienza University-Hospital, 10100 Turin, Italy; silvia.gaia74@gmail.com (S.G.); emanurolle@inwind.it (E.R.); daniela.campion@gmail.com (D.C.); paolabrunocilla@gmail.com (P.R.B.)

**Keywords:** SARS-CoV-2, ultrasound, alcohol, multifocal, nodule, Europe, cirrhosis, HBV, HCV, NAFLD

## Abstract

The COVID-19 pandemic has forced us to direct most of the available resources towards its management. This has led to the neglect of all other pathologies, including cancer. The aim of this study was to verify whether the difficulty in accessing the health system has led to a reduction in new diagnoses of hepatocellular carcinoma (HCC) and whether this has already been reflected in a more advanced stage of the cancer. A single-center, retrospective study including adult patients with a new diagnosis of HCC was performed. Patients were divided into three groups: the prelockdown phase (May 2019–February 2020), the lockdown phase (March 2020–December 2020), and the postlockdown phase (January 2021–October 2021); 247 patients were included. The number of patients diagnosed with HCC distinctly diminished in the periods March 2020–December 2020 (*n* = 69; −35%) and January 2021–October 2021 (*n* = 72; −32%) as compared to the period May 2019–February 2020 (*n* = 106). Noteworthy was the reduced surveillance in the period January 2021–October 2021 as compared to May 2019–February 2020 (22.9% vs. 36.6%, *p* = 0.056). No significant changes have yet been observed in tumor characteristics (BCLC staging distribution remained unvaried, *p* = 0.665). In conclusion, the number of new HCC diagnoses decreased sharply in the first 2 years of the pandemic, with no worsening of the stage. A more advanced stage of the disease could be expected in the next few years in patients who have escaped diagnosis.

## 1. Introduction

The COVID-19 pandemic has hit our societies and forced us to radically change our priorities [1,2]. This was especially true during lockdown periods, when even access to hospitals was limited to urgent cases [3,4]. This has led to suboptimal and delayed management of all other diseases [5]. Oncological pathologies have been particularly affected, and delays have been documented in screenings, referrals, diagnoses, therapy, and follow-up [6].

Hepatocellular carcinoma (HCC) is one of the most common cancers worldwide, and it is also a primary cause of cancer-related death [7]. The World Health Organization (WHO) predicts that over 1 million people will die from HCC in 2030 [8]. The WHO estimates that 0.1% of the European population suffers from liver cirrhosis, with a calculated 170,000 deaths annually; HCC is responsible for 47,000 deaths annually [9]. In Italy, mortality from liver cirrhosis and HCC is approximately 8.1 and 8.8 per 100,000 inhabitants, respectively [10]. Ultrasound screening in patients at risk of HCC allows diagnosis at an early stage of disease with a beneficial impact on survival [11].

The organization of the management of liver cancer patients has certainly changed during the pandemic [12]. In an online survey in Asia updated in May 2020, it was reported that there has been a 26.7% decrease in HCC diagnoses compared to the prepandemic period [13]. On the other hand, therapeutic management of patients diagnosed with HCC in the early months of pandemic (2020) appears to have been maintained at an adequate level [14], but although these previous studies have been fundamental, they have significant limitations. Notably, no data were collected to demonstrate whether the COVID-19 pandemic had adverse effects on the stage of HCC at diagnosis.

From this, it can be inferred that the difficulty in accessing the health system has led to a decline in the number of new HCC diagnoses, but it is not yet known whether this has already had an impact on the disease stage.

The aim of our study was to compare the number of new diagnoses of HCC before the pandemic with that during the first and second years of the COVID-19 pandemic and to assess whether a more advanced stage of HCC can already be observed.

## 2. Materials and Methods

### 2.1. Patients

This retrospective study included adult patients (≥18 years old) with a new diagnosis of HCC, recruited from 1 May 2019 to 31 October 2021 at the Gastroenterology Unit “Città della Salute e della Scienza di Torino-Molinette Hospital”, Turin, Italy; this is the largest hospital and university facility in the city (and in the region), with about 1900 beds, and a regional reference center for HCC; the city of Turin has 7 other minor hospitals and a population of approximately 887,000 inhabitants.

Ultrasound surveillance of patients affected by liver disease was not carried out in our clinic but was carried out by hepatologists from other centers or general practitioners. As our hospital is a third-level center, we received patients with suspected or newly diagnosed HCC, mainly found during six-monthly ultrasound screening (slightly less than 40%) or occasionally found in known or unknown liver disease (approximately 45–50%) or in the course of hepatic decompensation (10–15%). Patients with relapse of previously diagnosed HCC or with other primary liver tumors (i.e., cholangiocarcinoma) or liver metastasis were excluded from the study.

For the purpose of the study, patients were divided into three groups according to the time of HCC diagnosis: the prelockdown phase (May 2019–February 2020), the lockdown phase (March 2020–December 2020), and the postlockdown phase (January 2021–October 2021). Furthermore, the number of patients referred to our clinic with a new diagnosis of HCC was recovered from 1 January 2011. A specific database has been created for the collection of demographic, clinical, virological, and biochemical information. The presence of cirrhosis was determined by liver biopsy, liver elastography (FibroScan, Echosens, Paris, France), and/or hepatic ultrasound features and endoscopic signs of portal hypertension [15,16]. The diagnosis of HCC was achieved by histological examination or by contrast-enhanced imaging methods showing the radiological hallmark of HCC (i.e., the combination of hypervascularity in late arterial phase and washout on portal venous and/or delayed phases), according to international guidelines [17,18]. HCC was classified according to Barcelona Clinic Liver Cancer (BCLC) staging system (0 = very early; A = early; B = intermediate; C = advanced; D = end-stage) [17].

Study procedures were compliant with the principles of the Declaration of Helsinki. All patients gave their written informed consent, and the study was approved by the Ethics Committee of the Città della Salute e della Scienza—University Hospital of Turin (approval code 2CEI-452; 6 June 2012).

### 2.2. Statistical Analysis

Continuous variables were reported by median and interquartile range (IQR) while categorical variables were reported as number (*n*) and percentage (%). Data normality was checked by the D’Agostino–Pearson test. Comparison between unpaired groups was performed by the Mann–Whitney test for continuous variables and by the chi-squared (χ^2^) test for categorical variables, while comparison of continuous variables between more than two groups was performed by Kruskal–Wallis test. Linear regression analysis was performed to analyze the trend of the number of HCC diagnoses over time.

Since there is no study in the literature comparing the number of new diagnoses of HCC during lockdown, after lockdown, and before the COVID-19 pandemic, it was not possible to calculate the sample size in advance.

A two-tailed *p* value < 0.05 was considered statistically significant. All the statistical analyses were performed using MedCalc software, version 18.9.1 (MedCalc bvba, Ostend, Belgium).

## 3. Results

### 3.1. Characteristics of the Study Cohort

Overall, 247 patients with a new diagnosis of HCC were included in the study (from 1 May 2019 to 31 October 2021). The median age was 64.0 (56.0–71.8) years, and the patients were predominantly males (*n* = 199; 80.6%). The main underlying liver disease etiology was viral (*n* = 142; 57.5%) and the prevalence of cirrhosis was 96.4% (*n* = 238); the majority (*n* = 179; 75.5%) had a compensated liver disease (Child–Pugh score A). In most patients (*n* = 224; 91.4%), the diagnosis of HCC was obtained by imaging methods. Regarding tumor characteristics, 145 (58.7%) patients were diagnosed with an early tumor (BCLC 0/A); extrahepatic spread was detected in eight (3.2%) patients.

The principal demographic, clinical, and biochemical characteristics of the patients included in the study are reported in Table 1.

### 3.2. Comparison between the Different Periods of Enrollment

**Figure 1:**
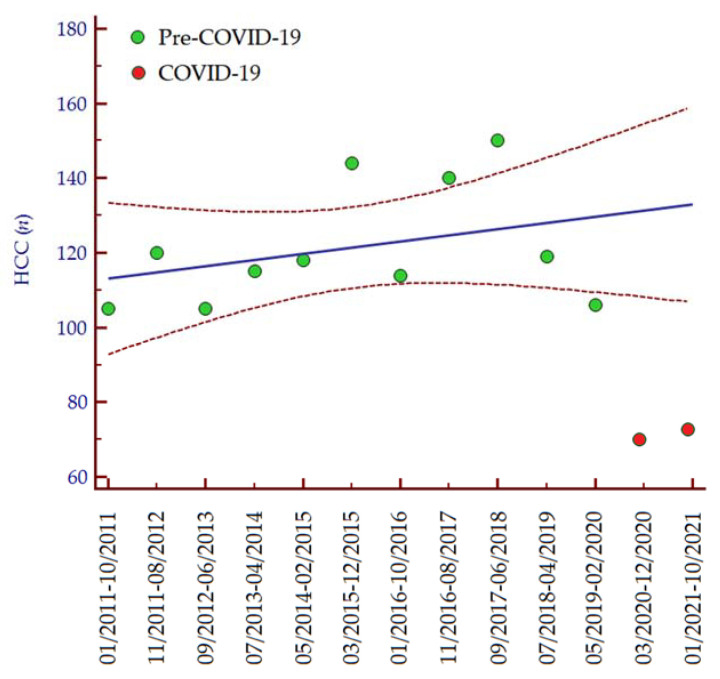
Number of HCC diagnoses per 10-month interval from 1 January 2011 to 31 October 2021. Regression equation: *y* = 111.527 + 0.165 ∗ *x*. The regression line is depicted in blue, while the 95% confidence interval of the regression line is depicted in red. Green dots indicate the number of patients diagnosed with HCC between January 2011 and February 2020, while the red dots indicate the number of patients diagnosed with HCC from March 2020 to October 2021. Abbreviations: coronavirus disease 19 (COVID-19), hepatocellular carcinoma (HCC), number (*n*).

The number of patients diagnosed with HCC distinctly diminished in the periods March 2020–December 2020 (*n* = 69; −35%) and January 2021–October 2021 (*n* = 72; −32%) as compared to the period May 2019–February 2020 (*n* = 106; 100%). Consistently, analyzing data from 1 January 2011, we observed an increasing trend in the number of HCC diagnoses per 10-month interval until the COVID-19 pandemic (Figure 1), while in the last two periods the number of HCC diagnoses was lower than expected (Table 2). In accordance with these data, the median number of HCC diagnoses in the last two periods (March 2020–November 2021) was significantly lower than the median number of HCC diagnoses per 10-month interval since the COVID-19 pandemic (January 2011–February 2020) (median 70, IQR 60–72, vs. median 118, IQR 105–150, *p* = 0.030, respectively).

Regarding characteristics of patients, we observed a stepwise increase in the prevalence of males over time, from 74.5% in the period May 2019–February 2020 to 82.6% in the period March 2020–December 2020 and 87.5% in the period January 2021–October 2021 (*p* = 0.029). Interestingly, BMI significantly increased from May 2019–February 2020 to January 2021–October 2021 period (median 25.5, IQR 22.7–28.8 kg/m^2^ vs. median 26.9, IQR 23.8–30.5 kg/m^2^, *p* = 0.025, respectively).

No variation was observed in the prevalence of cirrhosis (*p* = 0.475); consistently, no significant changes were observed in liver function parameters (platelet count, *p* = 0.180; albumin, *p* = 0.446; total bilirubin, *p* = 0.382) and in Child–Pugh score (*p* = 0.968). Remarkably, we noticed an increase in dysmetabolic etiology of liver disease, with a prevalence of 17.0% in the period May 2019–February 2020 to a prevalence of 26.1% in the period March 2020–December 2021, to 40.3% in the period January 2021–October 2021 (*p* < 0.001). Noteworthy was the reduction in regular ultrasound surveillance reported by patients diagnosed with HCC in the period January 2021–October 2021 compared to those diagnosed in the period May 2019–February 2020 (22.9% vs. 36.6%, *p* = 0.056).

No significant changes have yet been observed in the tumor characteristics. The number of patients with unifocal vs. multifocal HCC has remained stable over time (*p* = 0.331), as has the median size of HCC nodules (*p* = 0.887); in agreement with these findings, BCLC staging distribution has remained unchanged (*p* = 0.665).

## 4. Discussion

The five-year survival rate for HCC remains around 20%, partly due to poor adherence to screening and subsequent diagnostic and therapeutic delay [19]. Restrictions on access to the healthcare system due to the COVID-19 pandemic priority are likely to have made early detection of new HCCs even less efficient.

The data in the literature regarding the new diagnoses of HCC during these 2 years of the COVID-19 pandemic are limited to the first months of 2020. In a study conducted in Austria, 28 patients were diagnosed with HCC in the first half of 2020. There was no difference in the number of diagnoses of HCC before and after the lockdown (14 vs. 14). After the lockdown, more patients were delayed in visits and imaging tests, but no information was available on the stage of HCC [20]. In France, a significant decrease in the rate of HCC patients referred for first diagnosis or treatment was observed in the first weeks of the pandemic, but no data on the effect of lockdown after months or years are known [21]. In a recent letter to the editor, authors found an increased prevalence of HCC in patients admitted to hospital in 2020 due to alcoholic hepatitis (OR = 1.19; 95% CI 1.08–1.32): these findings may indicate a decline in HCC outpatient diagnosis as a result of the COVID-19 outbreak, which has caused a delay or discontinuity in regular HCC surveillance [22].

Our study included a large sample (*n* = 247) of new HCC diagnosed in a third-level center from 1 May 2019 to 31 October 2021. The characteristics of patients diagnosed with HCC were analyzed in detail throughout the lockdown period (2020) and in the following year (2021). During the lockdown (March 2020–December 2020) and after the lockdown (January 2021–October 2021) we observed a clear (over 30% decrease) and statistically significant reduction (*p* = 0.030) in the number of patients referred to our clinic for the first diagnosis of HCC compared to prepandemic period (May 2019–February 2020). It should be noted that our clinic did not apply any restriction to patients’ access during the pandemic period.

We can hypothesize some possible reasons for the negative impact of COVID-19 on the diagnosis of patients with HCC. During the COVID-19 pandemic, the government imposed two restrictions: social separation and house confinement, both of which were widely followed. Hence, liver patients at increased risk of HCC may not have been able to reach our hospital for medical consultations or may have received unspecified general appointments without priority, and this probably mirrored the general situation in our region, entrusted to an oncology network.

The most important reason that could explain the reduction in the number of new diagnoses of HCC is the marked reduction in 6-month ultrasound surveillance in cirrhotic patients at risk of HCC (from 36.6% before the pandemic to 22.9% in the year following the onset of the pandemic), probably due to limited access to diagnostic tests.

Although a more advanced disease with a more severe prognosis is not yet evident (BCLC comparison → *p* = 0.707; no significant difference in the number of patients with unifocal vs. multifocal HCC → *p* = 0.227, as well as in the median size of HCC nodules → *p* = 0.675), it is likely that we will find it when we diagnose HCC in 30–40% of patients who escaped diagnosis in these 2 years.

In our study, the prevalence of cirrhotic patients was slightly higher than that expected from the literature data (about 80% [23]), but compatible with a cohort in which HCV is still the main cause [24], despite a progressive increase in NAFLD as a cause (or a contributing cause) of HCC (from 17% to about 40%). This figure reflects the prediction that NAFLD will be the leading cause of HCC over the next decade [25], but it appears to have even worsened perhaps due to social restrictions and home confinement that limited physical activity; furthermore, we may have been more sensitive to reporting NAFLD as a contributing cause: in fact, its increase is not proportional to the decrease in viral infection, but is added to them. Our work presents some limitations, mainly that it is a retrospective, with limited sample size, and single-center study, which limits the applicability of our results: a validation of our results in a multicenter setting is mandatory. Strengths of our study include the following: it is the first study that analyzed a large number of new HCCs diagnosed during the first and second years of the pandemic and that has thoroughly analyzed the characteristics of patients with HCC in these 2 years.

## 5. Conclusions

In conclusion, our study shows that the COVID-19 pandemic had a significant impact on the number of patients at risk of HCC who have been diagnosed with cancer, although this did not result in more advanced HCC at diagnosis. Since HCC is a slow-growing cancer with a tumor doubling time of approximately 6 months, we expect the clinical consequences of a missed diagnosis to be more evident in the long-term follow-up.

## Figures and Tables

**Table 1:** Characteristics of the patients included in the study according to the different periods of enrollment.

Variables	Overall	March 2020– December 2020	March 2020– December 2020	January 2021– October 2021	*p* Value ^A^	*p* Value ^B^	*p* Value ^C^	*p* Value ^D^
Patients (*n*)	247	106	69	72				
Age (years), median (IQR); (247)	64.0 (56.0–71.8)	64.0 (55.0–73.0)	63.0 (56.0–69.5)	64.5 (58.0–70.0)	0.600	0.439	0.316	0.850
Gender (M/F), *n* (%); (247)	199 (80.6%)	79 (74.5%)	57 (82.6%)	63 (87.5%)	0.029 ^	0.211	0.416	0.035 ^
BMI (kg/m^2^), median (IQR); (247)	26.1 (23.3–29.4)	25.5 (22.7–28.8)	26.1 (23.1–29.1)	26.9 (23.8–30.5)	0.074	0.429	0.170	0.025 ^
Etiology, *n* (%); (247) *								
HCV	116 (47.0%)	51 (48.1%)	34 (49.3%)	31 (43.1%)	0.539	0.881	0.461	0.508
HBV	34 (13.8%)	16 (15.1%)	6 (8.7%)	12 (16.7%)	0.880	0.213	0.158	0.778
Alcohol	96 (38.9%)	42 (39.6%)	27 (39.1%)	27 (37.5%)	0.781	0.948	0.843	0.776
NAFLD	65 (26.3%)	18 (17.0%)	18 (26.1%)	29 (40.3%)	<0.001 ^	0.147	0.075	0.001 ^
Other	19 (7.7%)	10 (9.4%)	8 (11.6%)	1 (1.4%)	0.069	0.647	0.014 ^	0.029
Viral etiology, *n* (%); (247)	142 (57.5%)	64 (60.4%)	40 (58.0%)	38 (52.8%)	0.600	0.752	0.538	0.316
Platelet count (×10^9^/L), median (IQR); (241)	126 (84–85)	125 (86–184)	113 (74–181)	141 (103–193)	0.180	0.275	0.073	0.312
Albumin (g/dL), median (IQR); (190)	4.0 (3.4–4.4)	3.8 (3.3–4.4)	4.1 (3.5–4.4)	4.0 (3.4–4.3)	0.446	0.240	0.658	0.409
Total bilirubin (mg/dL), median (IQR); (232)	1.0 (0.7–1.7)	1.0 (0.6–1.6)	1.2 (0.7–1.8)	1.0 (0.7–1.9)	0.382	0.173	0.720	0.400
AFP (ng/mL), median (IQR); (196)	7.6 (3.3–101.5)	5.8 (3.6–122.6)	8.1 (2.8–105.7)	9.2 (3.1–75.1)	0.930	0.843	0.787	0.737
Cirrhosis, *n* (%); (247)	238 (96.4%)	101 (95.3%)	67 (97.1%)	70 (97.2%)	0.475	0.550	0.966	0.515
Semestral surveillance, *n* (%); (238) **	77 (32.4%)	37 (36.6%)	24 (35.8%)	16 (22.9%)	0.070	0.915	0.097	0.056
Child–Pugh score, *n* (%); (237)					0.968	0.777	0.922	0.925
A	179 (75.5%)	78 (77.2%)	48 (72.7%)	53 (75.7%)				
B	52 (21.9%)	21 (20.8%)	16 (24.2%)	15 (21.4%)				
C	6 (2.5%)	2 (2.0%)	2 (3.0%)	2 (2.9%)				
HCC diagnostic test, *n* (%); (245)					0.692	0.462	0.970	0.454
Liver biopsy	21 (8.6%)	8 (7.6%)	6 (8.7%)	7 (9.9%)				
Magnetic resonance	76 (31.0%)	28 (26.7%)	24 (34.8%)	24 (33.8%)				
Computed tomography	148 (60.4%)	69 (65.7%)	39 (56.5%)	40 (56.3%)				
HCC nodules, *n* (%); (247)					0.331	0.105	0.722	0.227
1	147 (59.5%)	56 (52.8%)	43 (62.3%)	48 (66.7%)				
2	31 (12.6%)	15 (14.2%)	11 (15.9%)	5 (6.9%)				
3	27 (10.9%)	14 (13.2%)	7 (10.1%)	6 (8.3%)				
>3	42 (17.0%)	21 (19.8%)	8 (11.6%)	13 (18.1%)				
Major HCC nodule (mm), median (IQR); (244)	29.5 (18.0–42.5)	27.0 (16.5–45.5)	28.0 (20.0–39.3)	30.0 (18.0–50.0)	0.887	0.933	0.657	0.675
Extrahepatic spread, *n* (%); (247)	8 (3.2%)	3 (2.8%)	2 (2.9%)	3 (4.2%)	0.637	0.979	0.685	0.629
BCLC, *n* (%); (247)					0.665	0.375	0.642	0.707
0	44 (17.8%)	17 (16.0%)	12 (17.4%)	15 (20.8%)				
A	101 (40.9%)	44 (41.5%)	32 (46.4%)	25 (34.7%)				
B	50 (20.2%)	19 (17.9%)	14 (20.3%)	17 (23.6%)				
C	48 (19.4%)	25 (23.6%)	9 (13.0%)	14 (19.4%)				
D	4 (1.6%)	1 (0.9%)	2 (2.9%)	1 (1.4%)				

Numbers in brackets indicate patients with available data. ^A^ Comparison between the three groups; *p* values were calculated by Kruskal–Wallis test for continuous variables and by χ^2^ test for categorical variables. ^B^ Comparison between the May 2019–February 2020 and March 2020–December 2020 periods; *p* values were calculated by Mann–Whitney test for continuous variables and by χ^2^ test for categorical variables. ^C^ Comparison between the March 2020–December 2020 and January 2021–October 2021 periods; *p* values were calculated by Mann–Whitney test for continuous variables and by χ^2^ test for categorical variables. ^D^ Comparison between the May 2019–February 2020 and January 2021–October 2021 periods; *p* values were calculated by Mann–Whitney test for continuous variables and by χ^2^ test for categorical variables. ^ Statistically significant. * Patients could have more than one cause of chronic liver disease. Among patients with chronic HCV infection, 72 out of 116 (62.1%) achieved SVR to previous antiviral treatment. Among patients with chronic HBV infection, 23 out of 34 (67.6%) were under nucleoside or nucleotide analog therapy at the time of HCC diagnosis. ** Calculated in patients with a diagnosis of cirrhosis (*n* = 238). Abbreviations: alphafetoprotein (AFP), Barcelona Clinic Liver Cancer (BCLC), body mass index (BMI), female (F), hepatitis B virus (HBV), hepatocellular carcinoma (HCC), hepatitis C virus (HCV), international normalized ratio (INR), interquartile range (IQR), male (M), number (*n*), nonalcoholic fatty liver disease (NAFLD), ultrasound (US).

**Table 2:** Numbers of observed and expected diagnoses of HCC from 1 January 2011 to 31 November 2021.

Time Period	Observed HCC Diagnosis (*n*)	Gender (M/F)	Viral Etiology *n* (%)	HCV *n* (%)	HBV *n* (%)	Etiology * Alcohol *n* (%)	NAFLD *n* (%)	Other *n* (%)	Expected HCC Diagnosis (*n*) **
January 2011–October 2011	105	81/24	70 (66.7%)	59 (56.2%)	14 (13.3%)	37 (35.2%)	7 (6.7%)	7 (6.7%)	
November 2011–August 2012	120	95/25	87 (72.5%)	67 (55.8%)	26 (21.7%)	38 (31.7%)	6 (5.0%)	11 (9.2%)	
September 2012–June 2013	105	82/23	76 (72.4%)	65 (61.9%)	15 (14.3%)	33 (31.4%)	10 (9.5%)	5 (4.8%)	
July 2013–April 2014	115	86/29	84 (73.0%)	67 (58.3%)	17 (14.8%)	44 (38.3%)	11 (9.6%)	2 (1.7%)	
May 2014–February 2015	118	93/25	87 (73.7%)	72 (61.0%)	18 (15.3%)	38 (32.2%)	14 (11.9%)	6 (5.1%)	
March 2015–December 2015	144	111/33	114 (79.2%)	94 (65.3%)	27 (18.8%)	60 (41.7%)	14 (9.7%)	5 (3.5%)	
January 2016–October 2016	114	86/28	80 (70.2%)	70 (61.4%)	10 (8.8%)	32 (28.1%)	18 (15.8%)	5 (4.4%)	
November 2016–August 2017	140	114/26	93 (66.4%)	83 (59.3%)	16 (11.4%)	59 (42.1%)	24 (17.1%)	3 (2.1%)	
September 2017–June 2018	150	117/33	99 (66.0%)	81 (54.0%)	18 (12.0%)	62 (41.3%)	35 (23.3%)	9 (6.0%)	
July 2018–April 2019	119	93/26	85 (71.4%)	69 (58.0%)	16 (13.4%)	52 (43.7%)	28 (23.5%)	5 (4.2%)	
May 2019–February 2020	106	79/27	64 (60.4%)	51 (48.1%)	16 (15.1%)	42 (39.6%)	18 (17.0%)	10 (9.4%)	
March 2020–December 2020	69	57/12	40 (58.0%)	34 (49.3%)	6 (8.7%)	27 (39.1%)	18 (26.1%)	8 (11.6%)	131
January 2021–October 2021	72	63/9	38 (52.8%)	31 (43.1%)	12 (16.7%)	27 (37.5%)	29 (40.3%)	1 (1.4%)	133

* Patients could have more than one cause of chronic liver disease. ** Estimated by regression equation. Abbreviations: hepatocellular carcinoma (HCC), number (*n*).

## Data Availability

The data presented in this study are available upon request from the corresponding author.
